# The Basic Biology of PP2A in Hematologic Cells and Malignancies

**DOI:** 10.3389/fonc.2014.00347

**Published:** 2014-12-11

**Authors:** Dorien Haesen, Ward Sents, Katleen Lemaire, Yana Hoorne, Veerle Janssens

**Affiliations:** ^1^Laboratory of Protein Phosphorylation and Proteomics, Department Cellular and Molecular Medicine, University of Leuven, Leuven, Belgium; ^2^Gene Expression Unit, Department Cellular and Molecular Medicine, University of Leuven, Leuven, Belgium

**Keywords:** PP2A, subunit, inhibitor, tumor suppressor reactivation therapy, PP2A-activating drugs

## Abstract

Reversible protein phosphorylation plays a crucial role in regulating cell signaling. In normal cells, phosphoregulation is tightly controlled by a network of protein kinases counterbalanced by several protein phosphatases. Deregulation of this delicate balance is widely recognized as a central mechanism by which cells escape external and internal self-limiting signals, eventually resulting in malignant transformation. A large fraction of hematologic malignancies is characterized by constitutive or unrestrained activation of oncogenic kinases. This is in part achieved by activating mutations, chromosomal rearrangements, or constitutive activation of upstream kinase regulators, in part by inactivation of their anti-oncogenic phosphatase counterparts. Protein phosphatase 2A (PP2A) represents a large family of cellular serine/threonine phosphatases with suspected tumor suppressive functions. In this review, we highlight our current knowledge about the complex structure and biology of these phosphatases in hematologic cells, thereby providing the rationale behind their diverse signaling functions. Eventually, this basic knowledge is a key to truly understand the tumor suppressive role of PP2A in leukemogenesis and to allow further rational development of therapeutic strategies targeting PP2A.

## Introduction

With 518 kinases encoded by the human genome and up to 70% of all eukaryotic proteins undergoing phosphorylation on a Ser, Thr, or Tyr residue, nearly every cellular process is controlled by this key modification (1). The covalent attachment of the bulky, negatively charged phosphoryl moiety to a protein markedly affects protein function through conformational changes that alter catalytic activity (for enzymes), affinity for ligands, subcellular localization, or stability (2). Several decades of biochemical and genetic studies have revealed crucial roles for protein kinases in the processes leading to tumor cell proliferation, survival, and migration in hematologic and other malignancies. In particular, genetic alterations that lead to constitutive activation of kinases, uncoupled from extracellular regulatory inputs, are well-characterized drivers of cancer development, a knowledge, which has emerged in the development of small-molecule kinase inhibitors for anti-cancer therapy (3). Kinase inhibitors have been extremely successful in the treatment of cancers driven by a single oncogenic kinase, such as chronic myeloid leukemia (CML) (4), but several challenges remain, including the development of drug resistance, lack of inhibitor selectivity or efficacy, and difficulty in drug target validation, particularly in cancers that do not exhibit such oncogenic kinase addiction (5).

Obviously, because protein phosphatases antagonize the action of protein kinases, they should be considered as equally important players in maintaining the correct phosphorylation balance of a given protein. Nonetheless, persistent misconceptions regarding the supposed lack of specificity and regulation of protein phosphatases as opposed to protein kinases, have contributed to a general underestimation of their critical role in the regulation of signal transduction (6, 7). Hence, much less is known about their role in cancer development and progression. Research over the past decade has begun to highlight the importance of the tumor suppressive activities of protein phosphatases, which, upon functional inactivation, contribute to persistent kinase or oncogene activation, and perhaps even more importantly, to drug resistance development (8, 9). Therefore, protein phosphatases may represent valuable novel drug targets for alternative cancer therapies, either in their own right or as part of combination therapies with kinase inhibitors (10–13).

Protein phosphatase 2A (PP2A) represents the prototype of a highly regulated phosphatase family with suspected critical tumor suppressive properties in several human tissues (14–16). Recent reports have demonstrated that modulation of PP2A activity can be beneficial for the treatment of cancer, particularly of hematologic malignancies (17, 18). Increasing evidence from cellular and clinical studies has indeed underscored the tumor suppressive role of PP2A in leukemogenesis, although the complex biology of these enzymes in hematologic cells remains incompletely understood. Here, we will provide insights into the basics of PP2A structure and regulation in hematologic cells and tissues, and highlight how proper PP2A function or activity is affected in hematologic malignancies. This knowledge is not only imperative to understand the protective role of PP2A in leukemogenesis but also equally important to allow for rational design of PP2A-directed drugs, and thus, to fully exploit PP2A as anti-cancer target in these devastating diseases.

## PP2A Family

### PP2A enzymes: Structural and functional centipedes

**Figure 1:**
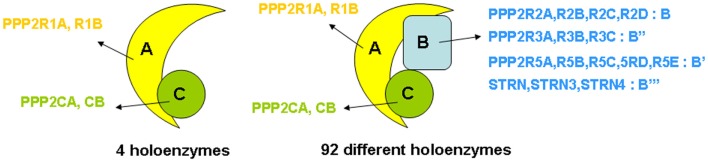
**Structure of PP2A holoenzymes**. The majority of PP2A enzymes have a heterotrimeric structure and consist of one catalytic C subunit, one scaffolding A subunit, and one regulatory B-type subunit. Owing to the existence of various isoforms of each of these subunits – in human tissues, two C (encoded by *PPP2CA* and *PPP2CB*), two A (encoded by *PPP2R1A* and *PPP2R1B*), and 23 B-type isoforms (encoded by 15 different genes) – 92 different PP2A trimeric complexes can be assembled, each characterized by its own catalytic properties, substrate specificities, tissue or cell-specific expression, and subcellular localization. In addition, about one-third of PP2A occurs as a dimer of one A and one C subunit (four holoenzymes).

“PP2A” refers to a large, highly conserved family of ubiquitously expressed Ser/Thr phosphatases that, together with PP1, constitutes the bulk of Ser/Thr phosphatase activity in a given cell or tissue (19). The prototypic PP2A holoenzyme is a heterotrimeric complex of a catalytic C subunit, a scaffolding A subunit, and a regulatory B-type subunit (Figure 1). In human cells, B-type subunits are encoded by 15 different genes, which give rise to 23 different isoforms through use of alternative gene promoters, alternative splicing events, or alternative translation (20). Based on sequence homology, they are divided into four distinct families, called B (or B55, or PR55, or by gene name: *PPP2R2*), B′ (or B56, or PR61, or by gene name: *PPP2R5*), B″ (or PR72, or by gene name: *PPP2R3*), and B‴ (or the striatins, *STRN*) (Figure 1). The B-type subunits are true “regulatory” subunits, in the sense that they dictate substrate specificity of the associated PP2A C subunit and can directly modulate PP2A catalytic activity. They are often expressed in a cell- or tissue-specific way, and can be found at distinct subcellular locations (cytoplasm, nucleus, plasma membrane, mitochondria, Golgi apparatus, endoplasmic reticulum, and cytoskeleton), thus, restricting PP2A activity to cell- or tissue-specific substrates present at specific subcellular sites (20, 21). Also, the C and A subunits are encoded by two different genes each, giving rise to two nearly identical Cα and Cβ isoforms (encoded by *PPP2CA* and *PPP2CB*), and two highly related Aα and Aβ isoforms (encoded by *PPP2R1A* and *PPP2R1B*). Despite an extremely high degree of sequence identity, there is evidence that these isoforms, remarkably, do not serve redundant functions (22–25). Besides their assembly into trimeric PP2A complexes, A and C subunits can form active A–C heterodimers (Figure 1), which are estimated to represent about one-third of cellular PP2A in a given cell (26).

The combinatorial assembly of one C and one A, or one C, one A, and one B-type subunit can theoretically give rise to 4 different heterodimers and 92 different heterotrimers (Figure 1), all exhibiting potentially different physiological functions. Thus, the broad diversity in PP2A composition creates specificity and constitutes the basis for the highly diverse and multiple cellular and physiological functions of these phosphatases. PP2A has indeed been implicated in a wide range of signaling pathways, many of which are involved in the control of cell proliferation and death (16, 27), cell division (28, 29), differentiation (28), adhesion and migration (30), and metabolism (31, 32). Besides function, PP2A composition also largely defines regulation by upstream factors, including specific second messengers (cAMP, Ca^2+^-ions, lipids) (20), cellular PP2A inhibitors (33) (see further), and phosphorylation by specific kinases (20). Most of these regulatory inputs are again largely determined by the nature of the specific B-type subunit present in the complex.

This being said, it should come of little surprise that “PP2A” (i.e., the large family of distinct PP2A complexes) may exert collaborating as well as opposing functions within a given signaling pathway by acting at different levels in the cascade. This is, for instance, the case in growth factor-induced ERK signaling, TGFβ signaling, or in canonical and non-canonical Wnt signaling (16, 27, 34). In addition, different PP2A complexes may dephosphorylate the same substrate, even on the same site, depending on the regulatory stimulus involved, the cell type or the broader physiological context (35–37). In contrast, functional redundancies, particularly between PP2A complexes harboring a B-type subunit from the same subfamily, have also been reported (38), further illustrating the complexity of PP2A holoenzyme function and substrate selection. It is clear though that in order to fully understand the role and regulation of “PP2A” in any (patho)physiological context, it is of utmost importance to identify which particular holoenzymes are involved in a non-redundant way. Nonetheless, and despite their general importance in PP2A biology, the specific PP2A regulatory subunits controlling dephosphorylation of a given substrate in a given mammalian cell or tissue remain poorly defined, particularly in the physiological context of a whole organism (39–41). Additional “PP2A” knockout mice are eagerly being awaited to overcome this lack of *in vivo* knowledge, and eventually, to advance the rational development of PP2A as a druggable target in the relevant cancer types.

### Expression of PP2A subunits in spleen, thymus, and bone marrow

**Figure 2:**
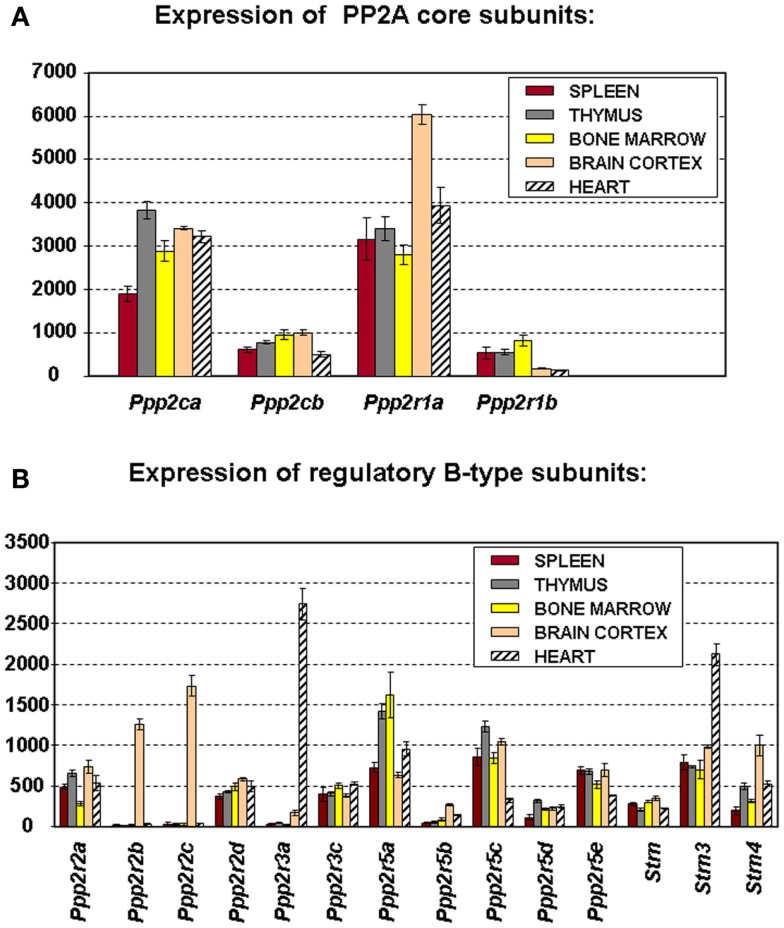
**Microarray expression profiles of PP2A subunit encoding genes in mouse tissues**. Spleen, thymus, bone marrow, brain cortex, and heart were hand-dissected from 10- to 12-week-old C57Bl6 mice. Total RNA was extracted, labeled, and hybridized to the Affymetrix mouse MOE 430 2.0 array (44). Scanning, quality control, data processing, and statistical analysis of the data were as described (44). Shown is the mean mRNA expression signal ±SD of three (spleen, thymus, brain, and heart) or four (bone marrow) biological replicate experiments. **(A)** Expression the PP2A core subunit encoding genes. **(B)** Expression of the genes encoding PP2A regulatory B-type subunits. Expression of *Ppp2r3d* could not be analyzed because it was not present on the array used.

To truly understand the biology of “PP2A” in hematologic cells and tissues, one should ideally know which PP2A complexes occur in these tissues. Because of general lack of sufficient isoform-specific antibodies and because only fragmented relevant information can be found in the currently available PP2A literature, we have analyzed, for the purpose of this review, mRNA expression of all PP2A subunit genes via microarray in mouse spleen (*n* = 3), thymus (*n* = 3), and bone marrow (*n* = 4) (Figure 2). Brain cortex (*n* = 3) and heart (*n* = 3) were included as “controls” (Figure 2) as in these tissues, expression of most PP2A subunits has been investigated and reported before. If we presume that hybridization of the PP2A mRNAs to their respective gene probes on the chip occurs with comparable efficiency, we find overall significantly higher expression of the α isoforms of both C and A subunits as opposed to their respective β isoforms (Figure 2A), fully in accordance with published data (19, 24). When considering expression of B-type subunits, most of them are expressed in all three hematologic tissues, except *Ppp2r2b* and *Ppp2r2c* (encoding Bβ and Bγ), which were reported to be exclusively expressed in brain (42), *Ppp2r3a* (encoding B″α), which was reported to be predominantly expressed in heart (43), and *Ppp2r5b* (encoding B′β) whose hematologic expression is extremely low (Figure 2B). Highest expression is seen for *Ppp2r5a* and *Ppp2r5c* (encoding B′α and B′γ), followed by *Ppp2r5e* (encoding B′ε), *Strn3* (encoding B‴/SG2NA), *Ppp2r3c* (encoding B″γ/G5PR), *Ppp2r2a*, and *Ppp2r2d* (encoding Bα and Bδ). Lowest expression is seen for *Ppp2r5d* (encoding B′δ), *Strn*, and *Strn4* (encoding B‴/striatin and B‴/zinedin) (Figure 2B). Expression of *Ppp2r3d* could not be analyzed because it was not represented on the microarray chip. For most PP2A subunits present in these tissues, expression is comparable between spleen, thymus, and bone marrow, except for *Ppp2r2a* (Bα), which is approximately two times more abundant in spleen and thymus as opposed to bone marrow, and for *Ppp2r5a* (B′α), *Ppp2r5d* (B′δ), and *Strn4* (B‴/zinedin), which are least abundant in spleen as opposed to thymus and bone marrow (Figure 2B). Thus, these data illustrate in a qualitative and semi-quantitative way the repertoire of PP2A B-type subunits expressed in the three main hematologic tissues in mice.

### Inactive PP2A complexes and PP2A holoenzyme assembly

**Figure 3:**
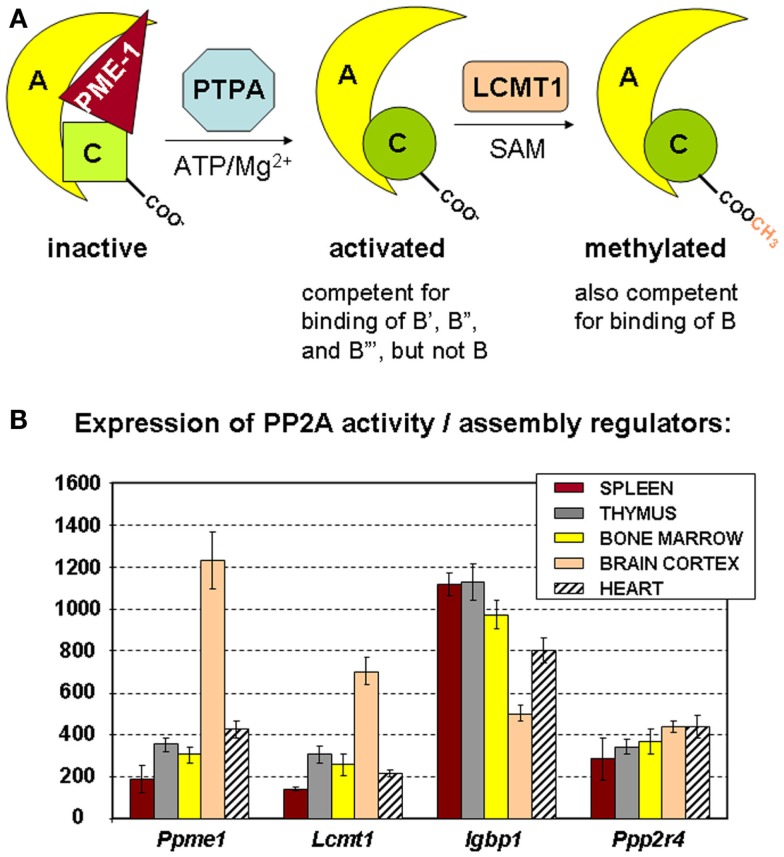
**Regulators of PP2A holoenzyme biogenesis and assembly**. **(A)** Simplified schematic of the roles of PME-1, PTPA, and LCMT1 in the biogenesis of active PP2A trimers. The PP2A methylesterase PME-1 serves to stabilize the inactive PP2A C subunit in a complex with the A subunit, at the same time preventing PP2A C methylation. With ATP/Mg^2+^ as necessary cofactors, PTPA promotes folding of PP2A C in an active conformation, and thereby, indirectly, PP2A C carboxymethylation by LCMT1. The latter modification is absolutely required for binding of B subunits, facilitates interaction of all B′ subunits but the δ isoform, is of no apparent importance for binding of B′δ and the B″ subunits, and is disliked by the striatin subunits. α4 (not depicted here) is another regulator that stabilizes PP2A C in a latent form. It is currently unclear if and how this inactive α4–C complex might become activated by similar mechanisms (47). **(B)** Expression of PP2A biogenesis regulators in hematologic tissues, brain, and heart. The mean mRNA expression signal ±SD for *Ppme1* (PME-1), *Lcmt1* (LCMT1), *Igbp1* (α4), and *Ppp2r4* (PTPA) is shown of three (spleen, thymus, brain, and heart) or four (bone marrow) biological replicate experiments.

Besides the prototypical PP2A holoenzymes described above, several “atypical” PP2A complexes have been identified that can occur within cells as catalytically inactive PP2A complexes. For example, the interaction between the C subunit and the α4 protein (encoded by *IGBP1*) stabilizes the C subunit as a latent, inactive form (45, 46), although there is also some evidence that this complex might be active toward very specific cellular substrates [reviewed in Ref. (47)]. Another example is the catalytically inactive complex between the C subunit, the A subunit, and PME-1 (PP2A Methyl Esterase 1, encoded by *PPME1*) (Figure 3A) that has been estimated to represent up to 25% of the cellular PP2A C pool (48, 49). It is thought that these atypical, inactive PP2A complexes constitute intermediate, but stable complexes during the process of PP2A holoenzyme biogenesis (47) or holoenzyme disassembly (45). Interestingly, increased expression of α4 or PME-1 has been found in several human cancers [hepatocellular carcinoma (50), lung carcinoma (50, 51), breast cancer (50), glioma (52), and endometrial cancer (53)], indicative for a relative increase in inactive PP2A complexes as opposed to active holoenzymes in these transformed cells.

The precise mechanism of assembly of active PP2A holoenzyme is still incompletely understood (47). A major insight came from the finding that the PP2A C subunit is synthesized/translated as an inactive enzyme (54) that is subsequently activated in a way that is strictly coupled to its incorporation into the complete holoenzyme (55). Like that, promiscuous and unregulated phosphatase activity of the free C subunit can be avoided (54, 55). There is evidence that proteins such as α4 and PME-1 can stabilize such inactive PP2A C subunits within cells, either in the absence (for α4) (46) or the presence (for PME-1) of the A subunit (47). To generate active PP2A holoenzymes from these inactive complexes, at least two additional PP2A regulating enzymes are needed. First, PTPA (or “PP2A Activator,” encoded by *PPP2R4*) may activate the PME-1-bound PP2A complex in the presence of ATP/Mg^2+^ as necessary cofactors (Figure 3A) (49). In accordance, *in vivo* data in yeast have shown that PTPA-dependent generation of active C subunit requires a functional interaction with the A subunit and is regulated by PME-1 (55). Crystallographic data suggested that PTPA may act as an ATP/Mg^2+^-dependent prolyl-peptidyl *cis/trans* isomerase of a single prolyl-peptidyl bond in PP2A C (56), as well as an ATP/Mg^2+^-dependent chaperone promoting the incorporation of catalytic metal ions into the PP2A C active site (57). Regardless of its precise mechanism-of-action, several *in vivo* studies have underscored the importance of PTPA as a physiological activator of PP2A [reviewed in Ref. (47)]. The second enzyme important in PP2A biogenesis is LCMT1 (leucine carboxyl methyl transferase 1, encoded by *LCMT1*), an *S*-adenosylmethionine-dependent methyltransferase catalyzing the carboxymethylation of the PP2A C subunit (58). This unusual post-translational modification of PP2A C is reversible through the presence of PME-1, the PP2A methylesterase (59), which may thus serve a dual function. Interestingly, PP2A C carboxymethylation requires an active PP2A C conformation (60), is facilitated by the presence of the A subunit (61), and enhances the affinity of the core dimer for PP2A regulatory subunits (Figure 3A). Specifically, PP2A C methylation is an absolute prerequisite to bind subunits of the B family, it facilitates interaction of all members but the δ isoform of the B′ family, is of no apparent importance for binding of B′δ and the B″ subunits, and is disliked by the B‴ subunits [reviewed in Ref. (62)]. Intriguingly, all regulators involved in PP2A holoenzyme biogenesis (α4, PME-1, PTPA, and LCMT1) are indispensable for mammalian survival (63–65, unpublished work), indicative for their physiological importance. In accordance, they are all expressed in spleen, thymus, and bone marrow, the three hematologic tissues analyzed here by microarray (Figure 3B). Our data also show a relatively higher expression of PME-1 and LCMT1 in brain, as opposed to other tissues analyzed (Figure 3B).

## Cellular PP2A Inhibitory Proteins

**Figure 4:**
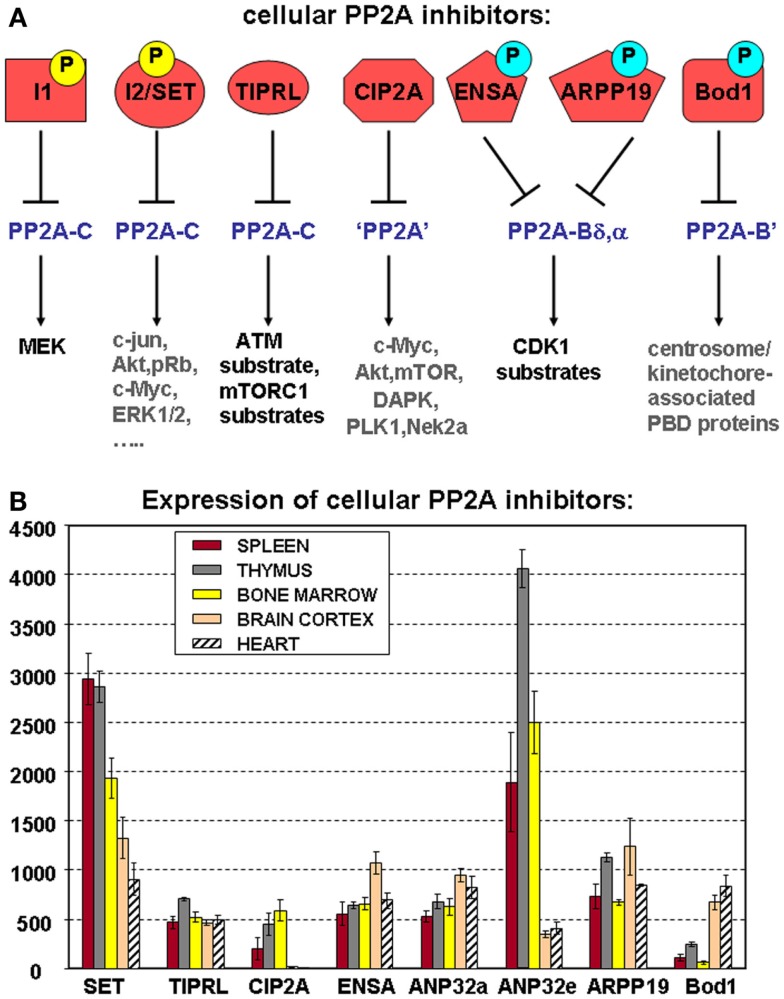
**Cellular PP2A inhibitors**. **(A)** Schematic representation of known cellular PP2A inhibitors, highlighting their potential regulation by (yellow) or dependence on (blue) phosphorylation, their holoenzyme specificity (if known), and the PP2A substrates they affect. Best characterized so far, in terms of phosphorylation dependence and holoenzyme specificity, are the mitotic inhibitors ENSA, ARPP-19, and Bod1. Both I1 (ANP32a, e) and I2 (SET) are established phosphoproteins, but depending on the specific site of modification, these phosphorylations may increase as well as decrease their PP2A inhibitory abilities. Phosphoregulation of CIP2A or TIPRL has not yet been described. I1, I2, and TIPRL are thought to interact with PP2A complexes through the C subunit, while holoenzyme specificity of CIP2A-mediated inhibition remains undefined. PBD: polo-box domain. **(B)** Expression of cellular PP2A inhibitors in hematologic tissues, brain, and heart. The mean mRNA expression signal ±SD for the eight indicated cellular PP2A inhibitory proteins is shown of three (spleen, thymus, brain, and heart) or four (bone marrow) biological replicate experiments.

Although the first cellular PP2A inhibitors were discovered almost two decades ago (66), their role in direct regulation of PP2A activity has only during the recent years come into considerable focus, not the least because some of them commonly suppress PP2A activity in hematologic and other cancers, and thus, may constitute novel therapeutic targets (33). These inhibitors either directly bind to the PP2A catalytic subunit or target very specific PP2A holoenzymes, thereby preventing dephosphorylation of a large variety of PP2A substrates (Figure 4A).

### ANP32a and SET

PP2A inhibitor 1 (also called ANP32a, or by gene name: *ANP32a*) and inhibitor 2 (also called TAF-Iβ, or PHAP1, or by gene name: *SET*) were originally *de novo* purified from bovine kidney as two potent, heat-stable PP2A-specific inhibitors (66) and subsequently cloned from cDNA libraries (67, 68). ANP32a belongs to a large family of at least nine members (ANP32a–h), of which only ANP32a and ANP32e show PP2A inhibitory ability in an *in vitro* phosphatase assay (69, 70). Free PP2A C subunit, the core A–C dimer and a trimeric PP2A complex with B subunit were inhibited in this assay, suggestive for direct binding of ANP32a to the C subunit, and thus, for no specific holoenzyme selectivity. Essentially, the same observations were made for SET in *in vitro* PP2A phosphatase assays (68). SET exists as two splice variants (SETα and β) that are both capable of inhibiting PP2A (71). ANP32a/e and SET are all phosphoproteins and can be found in the nucleus as well as the cytoplasm. Tyrosine phosphorylation of ANP32a releases its binding to PP2A and relieves PP2A inhibition toward MEK (72). In SETα, Ser9, Ser24, Ser93, and Ser171 have been identified as phosphorylation sites of functional importance (73–75). Phosphorylation of Ser9 functionally disrupts a nuclear localization signal and promotes SET retention in the cytoplasm (76, 77), while Ser171 phosphorylation decreases its PP2A inhibitory potential (74) and Ser9/Ser93 phosphorylation increases its ability to inhibit PP2A (75). Proteolytic cleavage of SET, either by Granzyme A at K176 (78) or by asparaginyl endopeptidase at N175 (79) is another mechanism that promotes its translocation into the cytoplasm (80), while the SET-binding protein SETBP1 stabilizes full-length SET inside the nucleus (81). Several PP2A substrates are known to be affected by SET, either directly or indirectly, including ERK1/2 (82), Akt (82–84), PTEN (83), Mcl-1 (85, 86), c-Myc (82, 84, 87), c-jun (88), and pRb (82), just to name a few (Figure 4A).

### CIP2A

CIP2A (or cancerous inhibitor of PP2A, or by gene name *KIAA1524*) is an oncoprotein, originally identified as a novel co-precipitating partner of the PP2A A subunit (89). CIP2A is barely detectable in normal cells, but becomes specifically upregulated in a large variety of human cancers, hence its name [reviewed in Ref. (90)]. CIP2A knockout mice show no overt phenotypes, except for a defect in spermatogenesis (91). In cancer cells, CIP2A upregulation is mediated by several oncogenic transcription factors, including Ets (92), Myc (93), and E2F (94), and often correlates with cancer aggressiveness and poor prognosis (90). At the signaling level, increased CIP2A expression has been associated with increased c-Myc stability and Ser62 phosphorylation (89), increased Akt signaling (95), inhibition of dependence receptor-dependent apoptosis (96), and more recently, with changes in regulation of cell cycle kinases such as Plk1 (97) and NEK2 (98), and activation of the TOR pathway (99, 100) (Figure 4A). The biochemistry of CIP2A remains, however, largely undefined; in particular, it remains to be determined which PP2A complexes it may specifically inhibit and how this is achieved.

### TIPRL1

TIPRL1 (also called TIP, or two A inhibitory protein, gene name *TIPRL*) is a ubiquitously expressed PP2A inhibitory protein that has been shown to inhibit free PP2A C and the PP2A A–C dimer by an allosteric mechanism (101, 102). TIPRL1 directly interacts with PP2A C, as well as with the C subunits of the PP2A-like phosphatases PP4 and PP6 (103). Notably, TIPRL1 may play an important role in DNA damage and repair signaling as it regulates PP2A enzymes that oppose ATM/ATR-dependent phosphorylation events (101). In addition, it may facilitate mTORC1 signaling and increase protein translation by sustaining phosphorylation of the mTORC1 substrates S6K1 and 4E-BP1 (104). In cancer cells, highly elevated TIPRL1 expression was reported in hepatocellular carcinoma, correlating with decreased pro-apoptotic MKK7/JNK signaling and contributing to resistance to TRAIL-induced apoptosis (105). The physiological role of TIPRL1 remains, however, undefined.

### Mitotic PP2A inhibitors: ENSA, ARPP-19, and Bod1

cAMP-regulated phosphoproteins ARPP-16 and ARPP-19 are splice variants and members of an evolutionary conserved protein family, to which ENSA (α-endosulfine) is closely related. ENSA and ARPP-19 are mitotic PP2A inhibitors that promote the G2/M transition and the mitotic state (106, 107). Intriguingly, they strongly bind to Bα and Bδ, but no other B-type subunits, dimeric PP2A or monomeric PP2A C, suggestive for a strong PP2A holoenzyme specificity (106–108) (Figure 4A). Moreover, these proteins require prior phosphorylation by the mitotic Greatwall/MASTL kinase, the Cdk1/cyclinB kinase, or potentially other mitotic kinases to exert their PP2A inhibitory effects (106, 107, 109, 110). Phosphorylation of ARPP-19 by cAMP-dependent kinase (PKA) serves to keep oocytes arrested in prophase (111), but how this may affect PP2A inhibition is unknown. More recently, yet another mitotic PP2A inhibitor was identified: Bod1, a protein required for proper chromosome alignment at mitosis. Bod1 shares sequence similarity with ENSA and ARPP-19, but intriguingly, specifically inhibits kinetochore- and centrosome-associated PP2A-B′ holoenzymes. Again, a phosphorylation of Bod1 by Cdk1/cyclinB is required to promote interaction with and inhibition of PP2A-B′ (112). Although many more needs to be discovered about the biochemistry and physiological roles of these mitotic PP2A inhibitors, they currently represent an exemplary mechanism of holoenzyme (family)-specific PP2A inhibition.

### Expression of PP2A inhibitors in hematologic tissues

As for the PP2A subunits (Figure 2) and the regulators of PP2A holoenzyme assembly (Figure 3B), we have analyzed mRNA expression of the above PP2A inhibitors in mouse spleen (*n* = 3), thymus (*n* = 3), and bone marrow (*n* = 4) (Figure 4B). Expression of SET, CIP2A, and ANP32e appears significantly higher in all three hematologic tissues analyzed, as opposed to terminally differentiated brain and heart tissues. In fact, CIP2A expression is completely undetectable in brain and heart, consistent with the idea that its expression is tightly coupled to cell proliferation, and potentially, stemness (90). Bod1 expression shows the opposite behavior and is significantly less expressed in spleen, thymus, and bone marrow as opposed to brain and heart, while TIPRL1, ANP32a, ENSA, and ARPP-19 expression is comparable in all tissues analyzed (Figure 4B).

## PP2A Aberrations in Hematologic Malignancies

Several mechanisms of PP2A dysfunction in hematologic malignancies have been reported, including changes in expression of PP2A subunits and inhibitors (by epigenetic or other mechanisms), genomic alterations in PP2A subunit and regulator encoding genes (including mutations, deletions, splicing errors, chromosomal translocations), and alterations in subunit modifications affecting PP2A activity.

### Alterations in PP2A subunits

Although both *PPP2R1A* (Aα) and *PPP2R1B* (Aβ) have been identified as genuine tumor suppressor genes in solid cancers (17, 25), few reports have currently documented their inactivation in hematologic malignancies. Decreased A subunit expression is observed in myeloid cells expressing activated c-KIT mutants (113), while loss of Aβ function occurs with low frequency in ALL (G90D mutation, 3/150) (114), B-CLL (exon skipping and reduced mRNA expression) (115, 116), and AML (117). Decreased expression of Cα is one of the hallmarks of del(5q) myelodysplastic syndromes (MDS) and AML, and interestingly, predicts a favorable therapy response to lenalidomide (118), suggestive for its use as a stratification marker. Increased Y307 phosphorylation of PP2A C occurred in 29/37 AML cases, correlating with significantly decreased PP2A activity toward Akt and ERK (117).

PP2A B-type subunit alterations occur more frequently, particularly in AML. Reduced expression of Bα in AML blasts, correlating with increased Akt, p70S6K, and PKCα phosphorylation and deregulated expression of specific microRNAs (miRs), is associated with significantly reduced complete remission duration (119, 120). In c-KIT mutant AML, reduced expression of Bα is observed, along with decreased expression of several B′ subunits (α,γ,δ), correlating with overall decreased PP2A activity (113). Genomic deletion of *PPP2R5B/C* (B′β,γ) (117) and downregulation of B′ε by an elusive non-genomic mechanism (121) do also frequently occur in AML, correlating with increased oncogenicity of the leukemic cells. In lymphocytic leukemia, *PPP2R5C* (B′γ) downregulation is a hallmark of progressive as opposed to stable B-CLL (122), while in Notch-induced T-ALL, *PPP2R5E* (B′ε) was identified as one of the targets for miR-19, an oncomiR that promotes leukemogenesis *in vivo* (123). In childhood T-ALL and B-ALL, *PPP2R3A* (B″α) is epigenetically inactivated by increased methylation with high frequency (69 and 82%, respectively) (124). Genomic deletion of *PPP2R2A/B* (Bα, β) is sporadically observed in primary plasma cell leukemia and multiple myeloma (125, 126).

### Alterations in PP2A regulators

The large majority of PP2A aberrations in hematologic malignancies involve abnormalities (overexpression, genetic modifications) in the proto-oncogenic PP2A inhibitors CIP2A and SET.

The first time deregulated CIP2A expression was linked to blood cancer development was through the discovery of a chromosomal translocation, resulting in an MLL-KIAA1524 fusion protein in an isolated case of infant AML (127). In this fusion, exons 1–10 of MLL are coupled in frame to exons 17–21 of CIP2A, encompassing the CIP2A coiled coil domain. In addition, CIP2A overexpression occurs frequently in newly diagnosed AML (54/70) and relapsed AML (11/14) (128). In CML, a positive feedback loop between CIP2A and BCR/ABL has been described, implying that CIP2A overexpression may promote CML pathogenesis (129, 130). Importantly, and in contrast to expression of SET, CIP2A expression is a clear determinant of disease progression to blast crisis (129) and thus confers a poor prognosis in these patients. Mechanistically, high-CIP2A levels in primary CML correlate with high levels of S62-phosphorylated c-Myc (129) and increased resistance to bortezomib-induced apoptosis (131). Analysis of CIP2A expression levels in a panel of 105 B-cell lymphomas further demonstrated a link with clinical aggressiveness of the subtypes, with weak or absent CIP2A expression in indolent B-cell lymphomas and strongly positive signals in the more aggressive diffuse large B-cell and Burkitt lymphoma subtypes (132).

Increased SET expression is found in CML, where it correlates with blast crisis and resistance to therapeutic BCR/ABL tyrosine kinase inhibitors (TKI) (82), in Philadelphia chromosome-positive (Ph)-ALL (133), (c-KIT positive) AML (113, 134), and B-CLL (85). In AML and B-CLL, its expression is associated with disease severity and poor outcome. In leukemic progenitors, PP2A activity is substantially impaired as a result of SET overexpression (82). In CML and Ph-ALL, induction of SET expression is controlled by BCR/ABL (82), while in AML, overexpression of EVl1 or downregulation of miR199b may contribute (134). Restoration of PP2A activity in leukemic cells results in decreased phosphorylation of pRb, c-Myc, Stat5, ERK1/2, Akt, Bad, and Jak2, and induction of SHP1-mediated BCR/ABL inactivation and degradation (82, 135). In atypical CML, lacking the BCR/ABL fusion, recurrent *SETBP1* mutations are found in 17/70 cases, some of which abrogate a site for ubiquitination, resulting in increased amounts of SETBP1 and SET protein, lower PP2A activity, and higher proliferation rates (136). In AML, overexpression of SETBP1 predicts poor outcome in elderly AML patients (81). Finally, *SET* is recurrently involved in chromosomal rearrangements and translocations, in particular, with the nucleoporin-encoding *Nup214* gene (also called *CAN*) in AML, T-ALL, and acute undifferentiated leukemia (137–140).

## PP2A (Re)Activation as a Novel Therapeutic Strategy in Hematologic Malignancies

The above findings, highlighting several mechanisms of PP2A inactivation in patients with hematologic malignancies, substantially underscore the tumor suppressor activities of (specific) PP2A holoenzymes and the proto-oncogenic properties of PP2A inhibitors CIP2A and SET. Importantly, some of these mechanisms may serve as biomarkers to improve current therapies (118), or may be directly amenable for therapeutic intervention. Several recent preclinical studies have shown that pharmacological restoration of PP2A tumor suppressor activity by PP2A-activating drugs (PADs) indeed effectively antagonizes cancer development and progression [reviewed in Ref. (17)]. Because PP2A complexes have so many cellular targets, these therapies may have the additional advantage, not to target just a single oncogene, but rather many different oncogenic pathways, contributing to their therapeutic efficacy. On the other hand, it is obvious that not all mechanisms of PP2A inhibition are suitable for restoration, particularly when subunit mutations are involved. Likewise, the development of small-molecule protein–protein interaction inhibitors targeting PP2A–SET or PP2A–CIP2A complexes remains, although attractive, extremely challenging.

Neviani et al. were the first to highlight the therapeutic relevance of using PP2A activators, such as FTY720 and forskolin, to target leukemia cells (82, 133). These observations prompted many others to test these compounds successfully in their own leukemic models (85, 113, 117, 141, 142). Treatment of AML patients with forskolin, in combination with standard induction therapy, gave an additive effect, highlighting therapeutic potential of PP2A activators in combination with standard chemotherapy (117). The mechanism of PP2A activation by these compounds remains somewhat obscure, but may involve direct binding of FTY720 to a ceramide-binding domain of SET (143, 144), resulting in SET dissociation from PP2A (143, 145). FTY720 also reduces SET Ser phosphorylation (144) and promotes SET nuclear localization (145), suggesting that its therapeutic effect may be largely attributable to restoration of cytoplasmic PP2A activity. The cell penetrating SET antagonistic peptides COG112 and OP449 (formerly COG449) directly bind SET to prevent SET–PP2A interaction and enhance PP2A activity (84). Like FTY720, they show significant therapeutic potential as PADs, as they induce apoptosis of human B-cell non-Hodgkin lymphoma and B-CLL *in vitro* and *in vivo*, without any discernable effects on normal B cells (85, 146). In models of human CML and AML (147) and canine T-cell lymphomas (148), OP449 also shows anti-tumoral effects, especially in combination with ABL TKI (147). The latter demonstrates the added benefit of combining TKIs and PADs for anti-leukemic therapy (17). Very recently, yet another class of FDA-approved drugs, the phenothiazines, were shown to act as PADs in models of T-ALL (149). These compounds induce rapid dephosphorylation of multiple PP2A targets, resulting in suppressed growth and increased apoptosis of T-ALL cells *in vitro* and *in vivo*. Mechanistically, a direct interaction with the Aα subunit is involved, but how this results in increased PP2A activity should still be further explored.

Together, these findings strongly encourage the inclusion of pharmacological PP2A activators with major anti-cancer activities and good safety profiles into current anti-cancer protocols in hematologic malignancies. The partially overlapping effects of existing drugs and PP2A stimulation predict that the inclusion of PADs in combination therapies with TKIs or other conventional therapeutics would represent particularly attractive therapeutic strategies to improve therapeutic outcome in these devastating malignancies. In the meantime, additional efforts to improve the potency and selectivity of existing PADs, and to identify alternative PP2A-activating strategies should be undertaken, in order to achieve their eventual use in the clinic.

## Conflict of Interest Statement

The authors declare that the research was conducted in the absence of any commercial or financial relationships that could be construed as a potential conflict of interest.
